# Characteristics of Mothers of Infants Born With Congenital Syphilis at a Tertiary Care Hospital in Fresno, United States

**DOI:** 10.7759/cureus.102736

**Published:** 2026-01-31

**Authors:** Richa Kaushal, Christine Nelson, Sandie Ha, Ratnali Jain, Jyothi R Patri

**Affiliations:** 1 Pediatrics, Fresno American Indian Health Project, Fresno, USA; 2 Pediatrics and Neonatology, University of California San Francisco, Fresno Regional Campus, Fresno, USA; 3 Public Health, University of California Merced, Merced, USA; 4 Biostatistics, University of California San Francisco, Fresno Regional Campus, Fresno, USA; 5 Family and Community Medicine, Lifestyle Medicine, Heritage Valley Health System Family Medicine Residency Program, Pittsburgh, USA

**Keywords:** barriers to healthcare access, congenital syphilis, drug use, inadequate prenatal care, inadequate treatment of syphilis, prenatal care, treatment of syphilis

## Abstract

Objective: Congenital syphilis (CS) continues to be a prevalent but preventable cause of infant morbidity and mortality. This study investigates the characteristics of women who delivered infants diagnosed with CS.

Methods: Retrospective data were collected from electronic medical records of 231 mother-newborn dyads diagnosed with CS at delivery in Community Regional Medical Center, a tertiary care hospital in Fresno, California, USA, between January 2010 and December 2018. T-tests and Fisher’s exact tests compared maternal and neonatal characteristics by syphilis treatment and prenatal care status. Log-linear models estimated relative risks (RRs) and 95% confidence intervals (CIs) for predictors of no prenatal care and no or inadequate treatment. All statistical tests were two-sided with an alpha level of 0.05.

Results: Twenty-one percent of women did not receive prenatal care, 51.5% received inadequate treatment, and 15% received no treatment for syphilis during pregnancy. No or insufficient syphilis treatment was more common among unemployed women, those who used drugs, smoked, had Medicaid, had no prenatal care, were incarcerated, or had abnormal neonatal outcomes. Drug use during pregnancy was associated with a 1.26-fold increased risk of receiving no or inadequate treatment (RR: 1.26, CI: 1.03-1.54) and a 3.32-fold increased risk (RR: 3.32, CI: 1.64-6.71) of having no prenatal care compared to women who did not use drugs.

Conclusion: Women from minority groups who were unmarried, unemployed, homeless, or using illicit drugs were more likely to receive inadequate prenatal care and no treatment for syphilis during pregnancy. Strengthening public health infrastructure to test and provide care for these high-risk pregnant women is urgently required.

## Introduction

Syphilis is a sexually transmitted infection (STI) caused by *Treponema pallidum*. Congenital syphilis (CS) occurs when this infection is vertically transmitted from mother to child transplacentally while pregnant and/or during delivery due to contact with maternal lesions or genital secretions. CS can result in various manifestations of varying severity, such as early fetal loss, stillbirth, premature birth, low birth weight, congenital disabilities, blindness, hearing loss, and even neonatal and infant death [1]. Penicillin G is the only known effective antimicrobial for treating syphilis in women, thereby preventing maternal transmission to the fetus. Penicillin is also the only drug that is used to treat CS. Based on the sexually transmitted disease (STD) treatment guidelines issued by the Centers for Disease Control and Prevention (CDC) in 2015, treatment of syphilis in pregnancy depends on the duration of infection. Primary, secondary, and early latent syphilis of less than one year should be treated with a single dose of 2.4 million units of benzathine penicillin G. In contrast, late, latent, or unknown duration of syphilis should be treated with benzathine penicillin G 2.4 million units once per week for three weeks. In pregnancy, there must be a strict seven-day gap between doses. One study evaluated the efficacy of treatment of syphilis with penicillin as recommended by the CDC and found that treatment with penicillin had a 98.2% success rate in preventing CS [2]. Adequate maternal treatment is defined as completion of a penicillin-based regimen recommended for the mother’s stage of syphilis initiated ≥30 days before delivery [3].

The United States Preventive Services Task Force and CDC recommend screening pregnant women for syphilis during their first prenatal visit. Screening again in the third trimester is promoted for women at increased risk of acquiring syphilis. [3,4] Even in areas where third-trimester screening is recommended, it has often been implemented at low rates. One study in Miami found that only 11% of women were re-screened in the third trimester, and another study from Texas found that only five of 31 providers routinely screened twice during pregnancy [5,6]. Despite the availability of testing and treatment, many pregnant women are not getting tested and are not receiving treatment for syphilis.

Based on the 2017 report by the CDC STD Control Branch, the United States has seen a surge in CS cases from 2011 to 2015. Trends in Western and Southern states drive national increases, with California accounting for approximately a third of the country’s total CS cases [7].

Fresno County, the setting of this study, is among the most affected counties in the United States for CS cases [8,9]. The county experiences high rates of poverty, drug use, STDs, and a shortage of healthcare workers. While previous research has primarily focused on urban settings, limited evidence is available regarding syphilis and CS in rural populations. This study aims to identify risk factors associated with inadequate prenatal care and insufficient treatment for syphilis among pregnant women in this context.

The objectives of this study are to describe the characteristics of women affected by syphilis during pregnancy and to investigate risk factors associated with inadequate prenatal care and insufficient treatment for syphilis.

## Materials and methods

A retrospective chart review was conducted at Community Regional Medical Center, a tertiary care 650-bed hospital in Fresno, California, USA. Mother-infant dyads with an International Classification of Diseases, Ninth Revision (ICD-9) or Tenth Revision (ICD-10) diagnosis of syphilis and a live-born infant delivered between January 1, 2010, and December 31, 2018 [10], were included, as shown in Table 1.

**Table 1:** International Classification of Disease (ICD) codes used to identify syphilis diagnoses. This table lists the International Classification of Diseases, Ninth Revision (ICD-9), and Tenth Revision (ICD-10), diagnosis codes used to identify maternal syphilis complicating pregnancy, childbirth, or the puerperium, as well as early and late congenital syphilis in neonates and children. These codes were used for case identification and classification in the study. Source: [10]

ICD-9 and ICD-10 Codes	Diagnosis
64700	Syphilis of mother, complicating pregnancy, unspecified episode of care
64701	Syphilis of mother, complicating pregnancy, delivered
64702	Syphilis of mother, complicating pregnancy, delivered with postpartum complication
64703	Syphilis of mother, complicating pregnancy, antepartum
64704	Syphilis of mother, complicating pregnancy, postpartum
098111	Syphilis complicating pregnancy, first trimester
098112	Syphilis complicating pregnancy, second trimester
098113	Syphilis complicating pregnancy, third trimester
098119	Syphilis complicating pregnancy, unspecified trimester
09812	Syphilis complicating childbirth
09813	Syphilis complicating the puerperium
A5009	Other early congenital syphilis, symptomatic
A501	Early congenital syphilis, latent
A502	Early congenital syphilis, unspecified
A5030	Late congenital syphilitic oculopathy, unspecified
A5031	Late congenital syphilitic interstitial keratitis
A5032	Late congenital syphilitic chorioretinitis
A5039	Other late congenital syphilitic oculopathy

Detailed data were collected regarding maternal and newborn characteristics. Maternal data were self-reporpted and included age, ethnicity (Caucasian, Hispanic, African American, Asian, American Indian, Mixed Ethnicity), insurance (Medical/Medicaid or Private), employment status (employed or unemployed), marital status (married, unmarried or divorced), incarceration (yes, no, unknown), presence of domestic violence (yes, no, unknown), homelessness (yes, no, unknown), smoking by mother (yes, no, unknown), alcohol use by mother (yes, no, unknown) drug use by mother (yes, no, unknown), type of drug use (methamphetamine, marijuana, opiates), other sexually transmitted infections during pregnancy (herpes simplex virus, chlamydia, gonorrhea, HIV), having prenatal care (yes, no), maternal treatment (adequate, inadequate, none, unknown), gestational age of baby at delivery (less than 32 weeks, 33-37 weeks, greater than 37 weeks), Group B Streptococcus screening status of mother (positive, negative, unknown), infant data collected included gestational age of baby at delivery (less than 32 weeks, 33 to 37 weeks, greater than 37 weeks), symptoms at delivery, and results of cerebrospinal fluid exam and treatment received.

Based on the Red Book of Pediatric Infectious Diseases, inadequate treatment for syphilis is defined as no treatment, undocumented treatment, treatment four weeks or less before delivery, treatment with a non-penicillin drug, or maternal evidence of reinfection or relapse (fourfold or greater increase in maternal rapid plasma reagin titers). Adequate treatment is defined as maternal penicillin treatment during pregnancy and more than four weeks before delivery, with no evidence or concern of maternal reinfection or relapse [11].

Statistical analysis

Frequency distributions were tabulated and examined for all variables, and data were reviewed for statistical outliers. T-tests and Fisher’s exact tests compared continuous and categorical maternal and neonatal characteristics, respectively, by syphilis treatment (adequate vs. no/inadequate) and prenatal care status (yes vs. no). Continuous predictors included maternal age, and categorical predictors included race/ethnicity, insurance status, marital status, prenatal care status, employment status, homelessness, domestic violence, incarceration status, drug use, smoking, alcohol use, STI status, gestational age, syphilis treatment status, and physical exam status in the newborn. Log-linear models estimated relative risks (RRs) and 95% confidence intervals (CIs) for significant predictors of no prenatal care and no/inadequate treatment. As the study did not aim for causal inference, only simple log-linear models were applied. All statistical analyses were performed using SAS Version 9.4 (SAS Institute Inc, Cary, NC, USA). Two-sided tests were used, and p-values < 0.05 were considered statistically significant.

## Results

Substance use was reported in 136 women (58.9%), including methamphetamine (48.5%), marijuana (24.7%), opiates (6.5%), smoking (33.8%), and alcohol (14%). Concomitant STDs included herpes simplex (26.4%), chlamydophila (15.2%), gonorrhea (4.8%), and HIV (2.6%). Herpes simplex infection was confirmed by a positive maternal IgM antibody test. Of the 231 women, 21% received no prenatal care, 51.5% received inadequate treatment, and 15% received no treatment for syphilis during pregnancy (Table 2).

**Table 2:** Characteristics of study participants (n=231) ᵃP-values were obtained from Fisher’s exact test for categorical variables, and t-tests for continuous variables (i.e., maternal age). Abbreviations: SD = standard deviation; CSF VDRL = cerebrospinal fluid Venereal Disease Research Laboratory test; GBS = Group B Streptococcus; HSV = herpes simplex virus; IM = intramuscular; IV = intravenous.

Characteristic	Category	All n (%)	Adequate Treatment n (row %)	No or Inadequate Treatment n (row %)	p-valueᵃ	Any Prenatal Care n (row %)	No Prenatal Care n (row %)	p-valueᵃ
Age (years)	Mean (SD)	27.3 (5.4)	26.3 (5.6)	27.8 (5.3)	0.039	27.0 (5.4)	27.9 (5.0)	0.274
Race/ethnicity	Caucasian	57 (25.2)	16 (28.1)	41 (71.9)	0.368	39 (68.4)	16 (28.1)	0.205
	Hispanic	142 (62.8)	49 (34.5)	91 (64.1)		106 (74.7)	27 (19.0)	
	African American	20 (8.9)	7 (35.0)	13 (65.0)		15 (75.0)	5 (25.0)	
	Asian	5 (2.2)	1 (20.0)	4 (80.0)		3 (60.0)	2 (40.0)	
	American Indian	1 (0.4)	1 (100)	0 (0)		1 (100)	0 (0)	
	Mixed ethnicity	1 (0.4)	0 (0)	1 (100)		1 (100)	0 (0)	
	Missing	5 (2.2)	1 (20.0)	3 (60.0)		3 (60.0)	0 (0)	
Health insurance	Medical/Medicaid	221 (95.7)	71 (32.1)	149 (67.4)	0.003			0.0002
	Private	4 (1.7)	2 (50.0)	2 (50.0)				
	Missing	6 (2.6)	2 (33.3)	2 (33.3)				
Marital status	Married	15 (6.5)	3 (20.0)	12 (80.0)	0.011			0.002
	Unmarried	205 (88.7)	68 (33.2)	136 (66.3)				
	Divorced	5 (2.2)	2 (40.0)	3 (60.0)				
	Missing	6 (2.6)	2 (33.3)	2 (33.3)				
Prenatal care	None	50 (21.7)	6 (12)	44 (88)	< .0001>			
	Any	168 (72.7)	60 (35.7)	106 (63.1)				
	Missing	13 (5.6)	9 (69.2)	3 (23.1)				
Employment	Employed	17 (7.4)	8 (47.1)	9 (52.9)	0.003	9 (52.9)	4 (23.5)	< .0001>
	Unemployed	207 (89.6)	64 (30.9)	142 (68.6)		156 (75.4)	46 (22.2)	
	Unknown	7 (3)	3 (42.9)	2 (28.6)		3 (42.9)	0 (0)	
Homeless	Yes	18 (7.8)	4 (22.2)	14 (77.8)	0.498	8 (44.4)	10 (55.6)	0.002
	No	77 (33.3)	29 (37.7)	48 (62.3)		60 (77.9)	16 (20.8)	
	Unknown	136 (58.9)	42 (30.9)	91 (66.9)		100 (73.5)	24 (17.7)	
Domestic violence	Yes	11 (4.8)	3 (27.3)	8 (72.7)	0.913	8 (72.7)	3 (27.3)	0.139
	No	50 (21.7)	18 (36)	32 (64)		42 (84)	8 (16)	
	Unknown	170 (73.6)	54 (31.8)	113 (66.5)		118 (69.4)	39 (22.9)	
Incarceration	Yes	17 (7.4)	9 (52.9)	8 (47.1)	0.001	14 (82.4)	3 (17.7)	0.001
	No	208 (90)	64 (30.8)	143 (68.8)		152 (73.1)	47 (22.6)	
	Unknown	6 (2.6)	2 (33.3)	2 (33.3)		2 (33.3)	0 (0)	
Drug use	Yes	136 (58.9)	36 (26.5)	98 (72.1)	0.002	91 (66.9)	42 (30.9)	< .0001>
	No	93 (40.3)	39 (41.9)	54 (58.1)		76 (81.7)	8 (8.6)	
	Unknown	2 (0.9)	0 (0)	1 (50)		1 (50)	0 (0)	
Methamphetamines	Yes	112 (48.5)	26 (23.2)	84 (75)	0.0003	37 (33)	73 (65.2)	< .0001>
	No	117 (50.7)	49 (41.9)	68 (58.1)		13 (11.1)	94 (80.3)	
	Unknown	2 (0.9)	0 (0)	1 (50)		0 (0)	1 (50)	
Marijuana	Yes	57 (24.7)	17 (29.8)	39 (68.4)	0.043			0.011
	No	172 (74.5)	58 (33.7)	113 (65.7)				
	Unknown	2 (0.9)	0 (0)	1 (50)				
Opiate	Yes	15 (6.5)	3 (20)	12 (80)	0.047	5 (33.3)	9 (60)	0.147
	No	214 (92.6)	72 (33.6)	140 (65.4)		45 (21)	158 (73.8)	
	Unknown	2 (0.9)	0 (0)	1 (50)		0 (0)	1 (50)	
Smoking	Yes	78 (33.8)	21 (26.9)	57 (73.1)	0.019			0.062
	No	151 (65.4)	54 (35.8)	95 (62.9)				
	Unknown	2 (0.9)	0 (0)	1 (50)				
Alcohol	Yes	14 (6.1)	6 (42.9)	8 (57.1)	0.052	9 (64.3)	5 (35.7)	0.165
	No	215 (93.1)	69 (32.1)	144 (67)		158 (73.5)	45 (20.9)	
	Unknown	2 (0.9)	0 (0)	1 (50)		1 (50)	0 (0)	
HSV	Yes	61 (26.4)	18 (29.5)	43 (70.5)	0.385	52 (85.3)	7 (11.5)	0.046
	No	28 (12.1)	13 (46.4)	15 (53.6)		22 (78.6)	6 (21.4)	
	Unknown	142 (61.5)	44 (31)	95 (66.9)		94 (66.2)	37 (26.1)	
Chlamydia	Yes	35 (15.2)	7 (20)	28 (80)	0.048	21 (60)	14 (40)	< .0001>
	No	155 (67.1)	58 (37.4)	96 (61.9)		126 (81.3)	23 (14.8)	
	Unknown	41 (17.8)	10 (24.4)	29 (70.7)		21 (51.2)	13 (31.7)	
Gonorrhea	Yes	11 (4.8)	1 (9.1)	10 (90.9)	0.053	6 (54.6)	5 (45.5)	0.0003
	No	178 (77.1)	64 (36)	113 (63.5)		141 (79.2)	31 (17.4)	
	Unknown	42 (18.2)	10 (23.8)	30 (71.4)		21 (50)	14 (33.3)	
HIV	Yes	6 (2.6)	1 (16.7)	5 (83.3)	0.025	5 (83.3)	1 (16.7)	0.016
	No	213 (92.2)	71 (33.3)	141 (66.2)		156 (73.2)	48 (22.5)	
	Unknown	12 (5.2)	3 (25)	7 (58.3)		7 (58.3)	1 (8.3)	
GBS status	Positive	82 (35.5)	31 (37.8)	50 (61)	0.164			0.001
	Negative	115 (49.8)	38 (33)	76 (66.1)				
	Unknown	34 (14.7)	6 (17.7)	27 (79.4)				
Mother treatment	Adequate	75 (32.5)				60 (80.0)	6 (8.0)	< .0001>
	Inadequate	119 (51.5)				93 (78.2)	25 (21.0)	
	None	34 (14.7)				13 (38.2)	19 (55.9)	
	Unknown	1 (0.4)				2 (66.7)	0 (0)	
Gestational age	<32 Weeks	19 (8.2)	2 (10.5)	16 (84.2)	0.005	13 (68.4)	6 (31.6)	0.049
	33-37 Weeks	57 (24.7)	12 (21.1)	45 (79)		36 (63.2)	19 (33.3)	
	> 37 Weeks	155 (67.1)	61 (39.4)	92 (59.4)		119 (76.8)	25 (16.1)	
CSF VDRL	Yes	113 (48.9)	21 (18.6)	91 (80.5)	< .0001>	79 (69.9)	32 (28.3)	0.01
	No	15 (6.5)	2 (13.3)	13 (86.7)		12 (80)	3 (20)	
	Unknown	103 (44.6)	52 (50.5)	49 (47.6)		77 (74.8)	15 (14.6)	
Physical exam in newborn	Normal	207 (89.6)	70 (33.8)	136 (65.7)	0.002	154 (74.4)	45 (21.7)	0.001
	Not normal	20 (8.7)	4 (20)	16 (80)		13 (65)	5 (25)	
	Unknown	4 (1.7)	1 (25)	1 (25)		1 (25)	0 (0)	
Treatment in newborn	Yes	220 (95.2)	69 (31.4)	150 (68.2)	0.0002	163 (74.1)	50 (22.7)	< .0001>
	No	7 (3)	5 (71.4)	2 (28.6)		4 (57.1)	0 (0)	
	Unknown	4 (1.7)	1 (25)	1 (25)		1 (25)	0 (0)	
Types of treatment in newborn	IM Penicillin	56 (24.2)	36 (64.3)	19 (33.9)	< .0001>	48 (85.7)	4 (7.1)	< .0001>
	IV Penicillin	139 (60.2)	25 (18)	114 (82)		95 (68.4)	42 (30.2)	
	both IM and IV	28 (12.1)	9 (32.1)	18 (64.3)		22 (78.6)	4 (14.3)	
	Unknown	8 (3.5)	5 (62.5)	2 (25)		3 (37.5)	0 (0)	

P-values were obtained from Fisher’s exact test for categorical variables, and t-tests for continuous variables (i.e., maternal age).

The proportion of women with no or inadequate syphilis treatment was 71.9% among Caucasian patients, 65.0% among African American patients, and 64.1% among Hispanic patients. Data for other groups were unstable due to small sample sizes. No or inadequate treatment was more common among women with Medical/Medicaid, those who were unmarried, lacked prenatal care, were unemployed, used illicit drugs, smoked, had concomitant Chlamydophila or HIV, or had a preterm delivery (Table 2).

Table 3 shows significant associations between maternal and neonatal outcomes and maternal prenatal and treatment status. Premature infants (<37 weeks) were more likely to be born to mothers with inadequate treatment. Women who used drugs, particularly methamphetamines and marijuana, were 1.26 times more likely to receive inadequate syphilis treatment (RR: 1.26, 95% CI: 1.03-1.54). Each additional year of maternal age was linked to a 1.0% increase in risk for insufficient treatment. Having an STI, such as chlamydophila or gonorrhea, was also associated with a higher likelihood of no or inadequate treatment. 

**Table 3:** Maternal and neonatal characteristics significantly associated with having inadequate treatment and no prenatal care ^a^Data are expressed as RR (95% CI). Relative risks were estimated using log-binomial regression models. Two-sided tests were used, and p-values < 0.05 were considered statistically significant. Abbreviations: RR = relative risk; CI = confidence interval; IM = intramuscular; IV = intravenous; NS = not significant.

Characteristic	Category	Inadequate Treatment RR (95% CI)^a^	No Prenatal Care RR (95% CI)^ a^
Maternal age (years)	Per year increase	1.01 (1.00–1.03)	NS
Homelessness	Yes	NS	2.64 (1.45–4.81)
	No	Reference	Reference
Drug use	Yes	1.26 (1.03–1.54)	3.32 (1.64–6.71)
	No	Reference	Reference
Methamphetamine use	Yes	1.31 (1.09–1.58)	2.77 (1.56–4.91)
	No	Reference	Reference
Marijuana use	Yes	NS	1.96 (1.22–3.16)
	No	Reference	Reference
Chlamydia	Yes	1.28 (1.04–1.58)	2.59 (1.49–4.51)
	No	Reference	Reference
Gonorrhea	Yes	1.42 (1.15–1.77)	2.52 (1.23–5.19)
	No	Reference	Reference
Gestational age	<32 weeks	1.48 (1.20–1.82)	1.82 (0.86–3.86)
	33–37 weeks	1.31 (1.09–1.58)	1.99 (1.20–3.31)
	>37 weeks	Reference	Reference
Type of maternal treatment	IM penicillin	Reference	Reference
	IV penicillin	2.37 (1.64–3.44)	3.99 (1.50–10.56)
	Both IM and IV	1.93 (1.23–3.03)	2.00 (0.54–7.36)

Women who used marijuana were 1.96 times more likely to have received no prenatal care. Those with concomitant chlamydia during pregnancy were 2.59 times more likely to lack prenatal care. Maternal age was not significantly associated with prenatal care. Women who used drugs had a 3.32-fold increased risk (RR: 3.32, 95% CI: 1.64-6.71) of having no prenatal care, especially those using methamphetamines and marijuana. Concomitant STDs, such as gonorrhea and chlamydophila, were also linked to inadequate prenatal care. Homeless women had 2.64 times the risk of no or insufficient prenatal care compared to those with housing (RR: 2.64, 95% CI: 1.45-4.81).

## Discussion

This study addresses a significant gap in the understanding of CS and corroborates the national trend of increasing CS cases since 2012. The retrospective chart review identified 231 mother-infant dyads diagnosed with syphilis during pregnancy and/or CS over nine years (Table 4). As shown in Table 4, the number of CS cases in Fresno County, California, has steadily increased since 2012. The findings demonstrate a strong association between inadequate prenatal care and insufficient treatment for syphilis.

**Table 4:** Number of congenital syphilis cases per year Source: [9]

Year of Birth	Frequency	Percentage of Live Births
2010	2	0.87
2011	3	1.30
2012	2	0.87
2013	16	6.93
2014	24	10.39
2015	41	17.75
2016	60	25.97
2017	45	19.48
2018	38	16.45

Although previous literature has examined risk factors for women delivering infants with CS, most studies have focused on urban populations. Mobley et al. investigated CS in a rural population in South Carolina and found that pregnant women with syphilis were more likely to deliver infants with CS than their urban counterparts [12]. Additional studies have identified racial and ethnic disparities in access to adequate prenatal care [13,14]. Slutsker et al. reported that 77.9% of women who delivered an infant with CS were Black or Hispanic women [15]. The present study demonstrates that other demographic and maternal characteristics are also statistically significant.

Studies show that CS is linked to lack of prenatal care, inadequate or no treatment, and a high rate of sexually transmitted diseases. Kidd et al. estimated that 75% of potential CS cases were successfully averted with proper prenatal care, testing, and adequate treatment [16]. A California study of missed opportunities to prevent CS identified gaps at multiple steps in the prevention cascade and found that early prenatal care is critical to preventing CS and that multifaceted efforts are needed [17].

The findings of this study are consistent with previous research and highlight the importance of adequate prenatal care and treatment in reducing CS cases. Li et al. demonstrated that programs implemented to prevent mother-to-child transmission of syphilis in Shanghai from 2001 to 2015 averted 39.4% of early fetal losses or stillbirths, 16.4% of neonatal deaths, 8.8% of prematurity or low birth weight, and 35.4% of CS cases [18]. Early prenatal care and STD testing are essential to protect women and infants from the severe consequences of this disease.

Future research should examine barriers to healthcare access, prenatal care, and immigration status. Further investigation is warranted to develop innovative strategies for patient motivation and education, implement community awareness campaigns, and provide prenatal care in sexually transmitted disease clinics. STD control programs should intensify efforts to identify women with STDs and drug use, ensuring these patients receive appropriate education.

Strategies that prioritize local community-level interventions to identify women at higher risk for maternal syphilis are essential. Identification, testing, treatment, and education constitute the foundation of this approach.

Case finding may be conducted at local drug rehabilitation centers, primary care offices, emergency departments, and hospitals. Care coordinators should be trained to track positive cases and facilitate referrals for appropriate treatment. States and localities with higher rates of CS should prioritize this issue due to the significant adverse outcomes for infants and children.

In states and counties with a higher incidence of CS, pregnancy testing should be routinely conducted alongside syphilis testing to facilitate early identification of syphilis cases. With sufficient funding, this testing could be expanded to all major sites in high-risk neighborhoods, potentially reducing CS incidence.

Mass awareness campaigns, conducted in collaboration with community partners serving vulnerable populations, can increase awareness of CS and emphasize prevention. Health policymakers, healthcare organizations, hospitals, and public health departments should collaborate to ensure that affected populations have access to care. A comprehensive and coordinated approach is required to reduce CS rates and improve maternal and child health outcomes. Sufficient financial resources are essential for the effective implementation of community outreach programs.

The primary strengths of this study include a large sample size for a rare condition, an extended study period, and comprehensive clinical and sociodemographic data collection from a high-burden rural setting. These factors enabled the inclusion of a robust cohort of CS cases. Nevertheless, the single-center, retrospective design restricts the generalizability of the findings to other populations. Furthermore, reliance on self-reported data, potential misclassification, and loss to follow-up among many women and their children limit the ability to draw definitive conclusions regarding long-term outcomes. A considerable proportion of women in this cohort did not receive prenatal care, which may confound the observed associations between maternal characteristics and treatment adequacy. Consequently, these results should be interpreted descriptively rather than as evidence of causality. 

## Conclusions

The study identified significant deficiencies in prenatal care and syphilis treatment among high-risk women residing in rural, underserved regions. The findings indicate that insufficient prenatal care and inadequate syphilis treatment during pregnancy are contributing factors to CS. Women from ethnic minority backgrounds who were unmarried, unemployed, homeless, or engaged in illicit drug use were disproportionately likely to receive inadequate prenatal care and no syphilis treatment, resulting in a higher likelihood of delivering infants with CS.

The study also identified key maternal factors linked to missed opportunities for prevention. Specifically, it characterized women who received no or inadequate prenatal care, which led to a lack of syphilis treatment during pregnancy. This subgroup delivered infants with CS in rural and underserved populations where the prevalence of syphilis and CS is rising annually. These results underscore the urgent need for targeted public health interventions to reduce CS rates and improve maternal and infant health outcomes.
